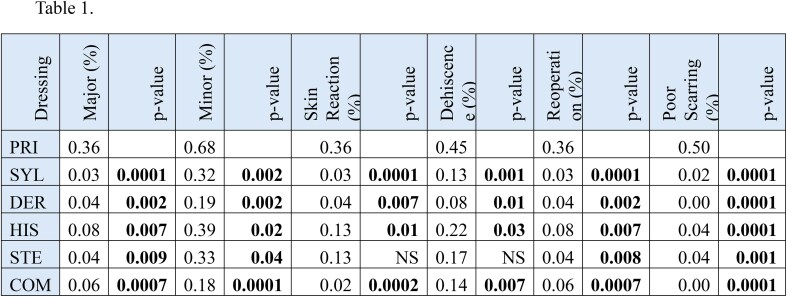# The Final Touch: Impact of Post-Operative Dressings on Reduction Mammoplasty

**DOI:** 10.1093/asjof/ojag138.019

**Published:** 2026-07-24

**Authors:** Steven Zeng, Thomas Ren, Ricardo Rodriguez, Nina Ringelman, Salman Choudhry, Gedge Rosson

**Affiliations:** Department of Plastic Surgery, Johns Hopkins Hospital, Baltimore, MD; Department of Plastic Surgery, Johns Hopkins Hospital, Baltimore, MD; Department of Plastic Surgery, Johns Hopkins Hospital, Baltimore, MD; Department of Plastic Surgery, Johns Hopkins Hospital, Baltimore, MD; Department of Plastic Surgery, Johns Hopkins Hospital, Baltimore, MD; Department of Plastic Surgery, Johns Hopkins Hospital, Baltimore, MD

## Abstract

**Goals/Purpose:**

Wound dressing is an essential step following reduction mammoplasty. An ideal dressing promotes healing, limits bacterial invasion, manages post-operative drainage, and is comfortable for the patient. Many dressing options, including surgical glues and adhesives, are currently available; however, no studies to date have identified a definitive, “ideal” dressing. This study aims to assess the impact on post-operative outcomes between commonly used post-operative dressings (Dermabond, Dermabond-Prineo, Histacryl, Sylke, and Steri-Strips) for reduction mammoplasty.

**Methods/Technique:**

A single-institution, IRB-approved review was conducted of patients undergoing reduction mammoplasty between July 2023 and July 2025. Patient demographics, operative details, post-operative dressings, and complications were collected. Cohorts were separated by post-operative dressing, which included Dermabond (DER), Dermabond Prineo (PRI), Histacryl (HIS), Sylke (SYL), Steri-Strips (STE), and a combination of multiple dressings (COM). Major complications included reoperation, and minor complications included skin reaction, hematoma/seroma, dehiscence, infection, need for antibiotics, nipple necrosis, and poor scarring. In patients undergoing an oncoplastic reduction or free-flap reconstruction, the contralateral, non-reduction breast was excluded. Student's t-tests for continuous variables and Pearson's chisquared test for categorical variables were used to evaluate the differences between groups. Multivariable logistic regression assessed the association of post-operative dressings with complications.

**Results/Complications:**

A total of 250 patients were identified, which included 439 breasts. Patients most commonly received Histacryl (38%) or Sylke (35%) as a post-operative dressing, followed by a combination of dressings (11%), Dermabond (6%), Dermabond Prineo (5%), and Steri-Strips (5%). The cohorts differed significantly in terms of BMI (p=0.0016), ASA class (p=0.001), diabetes (p=0.01), operative time (p=0.00001), and resection volume (p=0.00001). Of note, the Prineo cohort had higher rates of medical comorbidities, longer operative times, and larger resection volumes, and when excluded, there were no demographic or operative differences among the remaining cohorts.

Separate univariate analyses were conducted with and without the Prineo cohort. When included, Prineo had significantly higher rates of both major and minor complications among all groups. (HIS p=0.007, 0.02, SYL, p=0.0001, 0.002, COM, p=0.0007, 0.0001, DER, p=0.002, 0.002, and STE, p=0.009, 0.04). When assessing complications individually, Prineo was also noted to have higher rates of skin reactions, dehiscence, reoperation, and scarring when compared to other cohorts. (Table 1) When the Prineo cohort was excluded, Histacryl was found to have higher rates of skin reaction than Sylke (p=0.0029) and the combination cohort (p=0.049) and have higher rates of dehiscence than Sylke (p=0.043).

In the multivariate analysis, Sylke was predictive of lower rates of skin reaction (p=0.002, OR=0.2, 95%CI [0.06, 0.54]) and dehiscence (p=0.02, OR=0.47, 95%CI [0.25, 0.88]). Also, Prineo was predictive of higher rates of reoperation (p=0.01, OR=6.4, 95%CI [1.5, 28.5]) and poor scarring (p=0.0001, OR=34.8, 95%CI [6.1, 252.9]).

**Conclusion:**

Dermabond Prineo was associated with higher rates of skin reaction, wound dehiscence, poor scarring, and reoperation following reduction mammoplasty. Among the remaining dressings, Histacryl was associated with higher rates of skin reaction and dehiscence, and Sylke was predictive of lower skin reaction and dehiscence rates. However, these conclusions are potentially limited by confounding demographic and operative variables. Further, higher powered studies are also warranted to assess the impact of Dermabond and Steri-Strips in the setting of reduction mammoplasty.

**Figure:**